# Evaluation of the concept of students learning practical gynecological and obstetric skills at a large German university hospital, using the example of the Homburg university women's clinic

**DOI:** 10.1007/s00404-026-08473-5

**Published:** 2026-06-27

**Authors:** Sebastian Findeklee, Mariz Kasoha, Gregor Leonhard Olmes, Klaus Diedrich, Mihai-Teodor Georgescu, Romina Marina Sima, Erich-Franz Solomayer, Bashar Haj Hamoud

**Affiliations:** 1 Department for Gynecology, Obstetrics and Reproductive Medicine, Saarland University Hospital, Saarland University, Kirrberger Straße 100, Building 9, D-66421 Homburg, Germany; 2 MVZ Göttingen/Jena, Kasseler Landstraße 25a, D-37081 Göttingen, Germany; 3 Campus Lübeck, Frauenklinik, University Hospital Schleswig–Holstein, Ratzeburger Allee 160, D-23562 Lübeck, Germany; 4 Department for Obstetrics and Gynaecology, University Hospital “Carol Davila”, RO-020021 Bucaresti, Romania; 5 Saint John Hospital “Bucur” Maternity, RO-040294 Bucaresti, Romania

**Keywords:** Student teaching, Evaluation, Block internship, Gynecology and obstetrics

## Abstract

**Introduction:**

The teaching and practical training of future doctors play an important role in the university curriculum.

**Material and methods:**

At the Women’s Clinic of Saarland University Hospital in Homburg, we developed a five-day block placement concept that focused on learning practical gynecological and obstetric skills rather than simply imparting theoretical knowledge. The student`s satisfaction with the block placement was assessed using a non-standardized questionnaire with the two items “How do you rate your learning outcomes from the placement?” and “The placement increased my interest in gynecology and obstetrics”. The assessment was based on a scale of 1 to 6.

**Outcome:**

A total of 205 out of 220 students (93.2%) from the winter semester 2022/2023 participated in the evaluation and completed the questionnaire. 198 students (96.6%) rated the satisfaction with their perceived learning from the block internship as very good (1) or good (2), and 201 students (98%) reported an increase in their interest in the subject area.

**Discussion:**

An overwhelming majority of students rated the practical teaching concept as very good or good, which speaks to the success of this practical teaching format. Nevertheless, the study also has several limitations. For example, only one cohort of medical students was surveyed. Furthermore, when establishing a new teaching concept, there is a risk that participants in an evaluation will not respond objectively, but rather according to the criterion of social desirability. We suggest our practice-oriented block placement concept, which focuses on learning skills relevant to the future medical profession, as a possible curriculum for undergraduate teaching in gynecology and obstetrics, to inspire students for the field.

## Introduction

Student teaching is playing an increasingly important role at German university obstetrics and gynecology clinics. A key component of the training of future physicians in the field of obstetrics and gynecology is the clinical rotation. This rotation is mandatory in the curriculum and typically lasts 4–5 days, usually in the 4th or 5th year of study [1]. The main objective should be for future colleagues to gain a comprehensive overview of the field and to acquire practical skills alongside theoretical knowledge. Further objectives include, in the short term, preparation for the written and, if applicable, oral-practical second state examination in medicine (administered in multiple-choice format), and, in the long term, fostering motivation to work in obstetrics and gynecology in the future.

At the Saarland University obstetrics and gynecology clinic in Homburg, the clinical rotation is implemented by a designated instructor, the clinic's teaching coordinator. The rotation has been continuously developed over the course of the semesters [2]. Both student feedback and internal discussions within the teaching department proved helpful. Although each university women's hospital in Germany offers its own individual clinical rotation, to our knowledge there is no recognized standard for the practical procedure. No publication explicitly describes the process and could serve as an example for other hospitals.

Below, we describe the structure of the obstetrics and gynecology clinical rotation at the Homburg University Women's Hospital. We have had positive experiences with the procedure described here and have achieved a high level of satisfaction among the participating students.

## Material and methods

### Schedule of the block internship

The clinical placement at the University Women ‘s Hospital in Homburg lasts 5 days (Monday to Friday). It is divided into a clinical session in the morning from 8:00 a.m. to 12:30 p.m. followed—after a one-hour lunch break—by a seminar session from 1:30 p.m. to 4:00 p.m. Each week of the semester, a seminar group, typically consisting of 10 students, participates.

The clinical placement begins on Monday at 7:45 a.m. with a 15 min introductory session. During this session, the students are given an explanation of the schedule for the placement. Each student also receives a personalized name badge and a logbook for the placement. This logbook includes a schedule for the placement and a guide to fetometry in obstetric ultrasound. From 8:00 to 8:15 a.m., a tour of the clinic's five functional areas takes place. Typically, two students visit each area daily, depending on the size of the seminar group. These five areas are the general gynecology outpatient clinic with its specialized consultations for fertility, dysplasia, and urogynecology; the oncology outpatient clinic; the antenatal clinic; the delivery room; and the operating room. Following this, students provide an introduction to obstetric ultrasound for other students. During this 30-min session, a student tutor explains the fundamentals of ultrasound and the procedure for fetometry. At the end of each morning, the tutor takes the student assigned to the antenatal clinic to practice fetometry at the bedside of a pregnant patient. The patients are asked beforehand and must consent to participate. In the afternoon, following some general remarks about the field and the structure of the training, the procedure for a gynecological examination using a speculum and breast trainer is explained, employing a pelvic phantom (for nulliparous and multiparous women) and a breast phantom (Koken 14–3 Mejiro 3-Chome, BCS 6476). Students then practice the examination in pairs under supervision. This is followed by a cardiotocography (CTG) course, in which the fundamentals of CTG analysis are taught based on the four basic criteria: basal rate, bandwidth, decelerations, and accelerations. Each student then receives a CTG and must analyze it according to these four criteria. Monday afternoon concludes with a laparoscopy video demonstrating the intraoperative site and anatomical structures.

On Tuesday afternoon, the process of natural childbirth is discussed using a birth mannequin and fetal movements. Following this, the students demonstrate the previously demonstrated movements in pairs. Next, a role-playing exercise takes place in which the students must manage four obstetric emergencies in the delivery room (pathological CTG requiring emergency cesarean section, pathological CTG requiring vacuum extraction, shoulder dystocia, and uterine atony). The delivery room resident can then apply the CTG knowledge acquired the previous day. A subsequent discussion of the case and the emergency procedures follows.

On Wednesday afternoon, a conization course is held. Here, each student practices conization on a sausage using the self-developed Homburg conization model with a monopolar loop [3]. Success is assessed by measuring the cone thickness (target 7–10 mm). If necessary, a re-resection is performed. The second part of the afternoon seminar consists of a 15 min introduction to mammosonography. Then, each student, divided into two groups, performs mammosonography at the patient's bedside under supervision. The patient had to have previously agreed to participate voluntarily. Afterwards, there is an opportunity to practice a core needle biopsy of the breast using a self-made gelatin breast model with an olive-shaped tissue sample. The presence of a piece of the olive in the biopsy channel serves as confirmation of success.

Since the summer semester of 2018, on Thursday mornings, students have had the opportunity to attend the endoscopy school run by a lecturer at Saarland University Hospital in Saarbrücken, instead of the five functional areas of the clinic. Here, the fundamentals of gynecological endoscopy are taught, and students have the opportunity to practice their skills on models they have designed themselves. In the second part, advanced students practice using laparoscopy instruments on a pelvic model and perform laparoscopic suturing. A laparoscopy simulator developed specifically by us was used for this purpose [4]. In the afternoon, the three most common gynecological cancers in Germany (endometrial, ovarian, and cervical cancer) will be discussed interactively using a clear whiteboard diagram. This will be followed—as a synthesis of gynecological and obstetrical disease patterns and preparation for the final exam—by a case seminar on gynecological and obstetric emergencies. Here, each student will receive a case presented in a PowerPoint presentation and will be asked to identify possible differential diagnoses and describe the diagnostic procedure in order to determine the most probable diagnosis.

The final exam takes place on Friday afternoon. Each student presents a patient case with a specific condition (gestational diabetes, ectopic pregnancy, hypertensive disorder of pregnancy, miscarriage, uterine fibroids, breast cancer, ovarian cancer, cervical cancer, endometrial cancer, endometriosis). This condition is assigned to each participant at the beginning of the clinical rotation. Ideally, students should have seen the patient themselves during one of the mornings. If this is not possible, the ward physicians will provide assistance. The case presentation lasts approximately 5 min. This is followed by 2–3 questions about the condition, primarily for review and reinforcement within the group. The presentation is graded on a scale of 1–6 (German grading system).

**Fig. 1:**
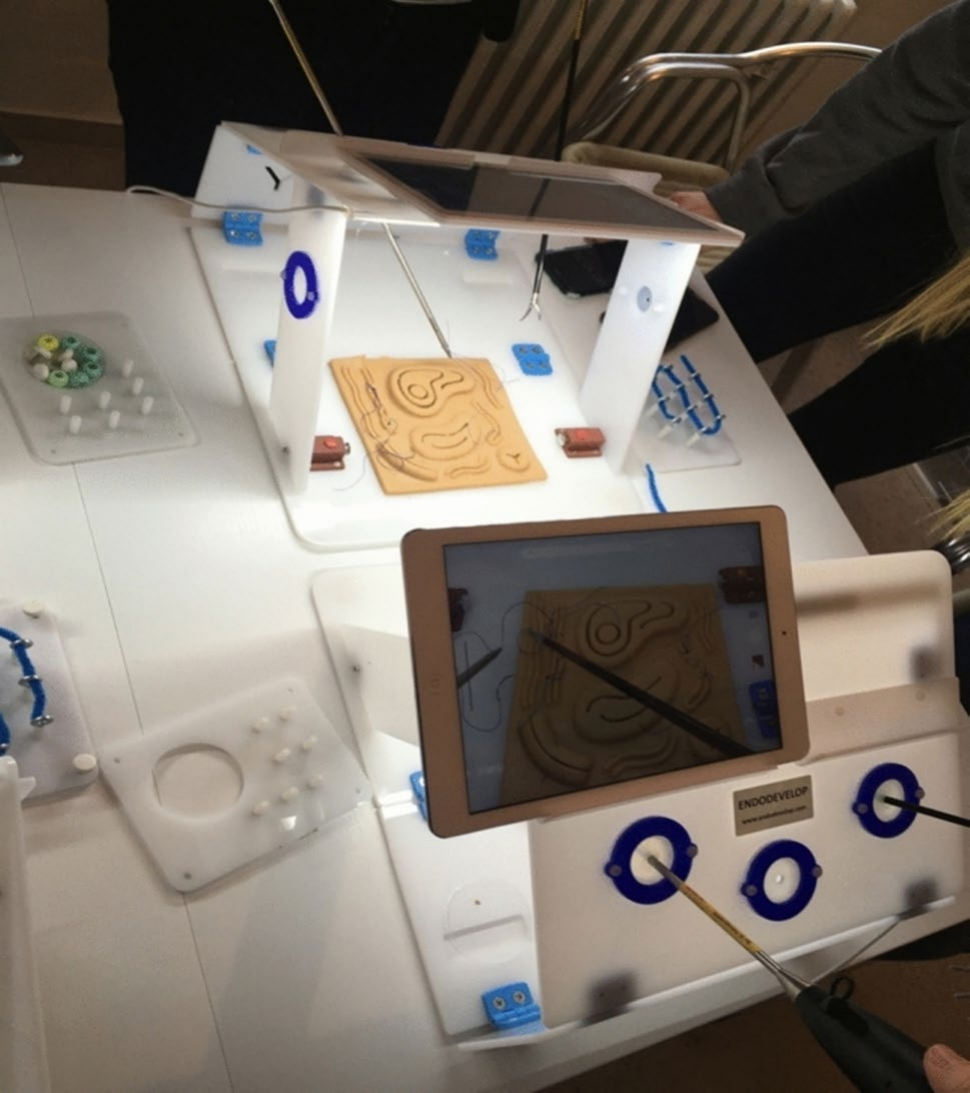
Homburg laparoscopy simulator

A supplementary script is available for the clinical rotation to further explore the material and prepare for the exam. Students can download this from the internet with password protection (Fig. 1).

### Evaluation of the student satisfaction with the block internship

We conducted a single-center, descriptive survey evaluating the acceptability and feasibility of a skills-based internship in gynecology and obstetrics. The evaluation instrument was a non-standardized evaluation form from Saarland University Hospital, consisting of two non-validated questions: “How do you rate the learning outcomes of the placement?” and "The placement has increased my interest in the field of gynecology and obstetrics." Students could rate their responses on a scale of 1 to 6 (see Table 1). The analysis was purely descriptive without statistical analysis. Ethical approval was requested from the Ethics Committee of the Medical Faculty of Saarland University. The Ethics Committee stated that an approval would not be necessary, since both teaching knowledge and imparting practical skills as well as its evaluation by the students are mandatory components of the student curriculum.

**Table 1:** results of the evaluation of the block internship in gynecology and obstetrics at the University Women’s Hospital Homburg

Item	very good	good	satisfactory	sufficient	inadequate	insufficient
How do you assess the learning *experience* of the internship?	166 (81%)	32 (15.6%)	4 (2%)	3 (1.4%)	0	0
The internship strengthened my interest in the field of gynecology and obstetrics	175 (85.4%)	26 (12.7%)	3 (1.4%)	0	0	1 (0.5%)

*legend

1- very good or applicable

2- good or mostly applicable

3- satisfactory or partially applicable

4- sufficient or rather not applicable

5- **i**nadequate or mostly not applicable

6- insufficient or not applicable

## Outcome

220 students completed the block placement in obstetrics and gynecology at the Homburg University Women's Hospital in the winter semester of 2022/2023. The course was evaluated anonymously by 205 students, representing 93.2% of the total.

166 students (81%) rated the satisfaction with their perceived learning as very good, 32 (15.6%) as good, 4 (2%) as satisfactory, and 3 (1.4%) as adequate. No students received a failing or unsatisfactory rating. 175 students (85.4%) agreed that the placement increased their interest in obstetrics and gynecology, 26 students (12.7%) stated that this was mostly true, 3 students (1.4%) found it partially true, and only one student (0.5%) stated that it was not true. The evaluation results are summarized in Table 1.

## Discussion

The obstetrics and gynecology block internship can justifiably be described as the heart of undergraduate medical education in our field. It undoubtedly represents a crucial moment in the curriculum, as it offers the opportunity of presenting the field in all its breadth and of inspiring future colleagues to pursue our specialization. It can also be seen as the final opportunity for self-promotion before choosing an elective rotation during the final year (practical year) of medical studies.

Various models designed for undergraduate medical education exist in the literature. These range from role-playing to practical exercises using models [5, 6]. These elements are also incorporated into our block placement. Integrating the greatest possible proportion of practical exercises into the placement was particularly important to us, as theoretical knowledge can also be acquired through self-study, for example, with the help of a provided accompanying script. At the same time, it was essential to also cover theory to meet the immediate goal of exam preparation. Therefore, following the introduction of the optional endoscopy course on Thursday mornings in the summer semester of 2018, we decided to offer a theory block in the afternoon. This clearly demonstrates that the clinical rotations at our clinic are subject to constant change. They are continuously developed in consultation with clinic colleagues and students. Only in this way can they fulfill their objectives: imparting specialist knowledge, teaching human qualities, such as empathy and communication skills, conveying principles of responsible medical practice, and, last but not least, inspiring young people to pursue a career in obstetrics and gynecology. This component appears particularly pressing in light of demographic trends.

A view of the literature reveals scattered publications from the USA regarding skill-based internships and training concepts for medical students [7, 8]. Despite its importance—both for the quality of clinical training and for the recruitment of future gynecologists—there is no recognized standard for the implementation of the clinical rotation among German university obstetrics and gynecology departments.

In 2025, Adams et al. published a simulation-based training program for medical students. Strengths of this study, compared to our survey, included the conduct of a pre/post assessment and the measurement of objective outcome parameters. Conversely, the number of participants in the intervention group—at 65—was relatively low. Duhm et al. demonstrated that clinical exposure and mentorship increase students' interest in the field of obstetrics and gynecology, which was also observed by us [9, 10].

Due to the significant practical components and the high acceptability in our survey we suggest the internship developed at our clinic as a possible curriculum for undergraduate teaching in gynecology and obstetrics, which could also be performed at other university hospitals. We base this proposal on our own positive experiences implementing it under everyday clinical conditions combined with a high level of student satisfaction. The positive evaluation results are consistent with previous studies from our department, which show, on the one hand, that students can acquire practical skills very quickly and, on the other hand, that they evaluate practical teaching very well [11, 12].

Strengths of our descriptive study are the clearly described intervention including a practical, reproducible teaching model and the high participation rate.

However, our survey has lots of limitations. Therefore, our findings have to be regarded with the highest caution. First of all, it is a single-center, descriptive survey with no control group and no pre/post assessment. Second, only a subjective outcome (student satisfaction) was measured. The evaluation is based on only two non-validated questions addressing student-rated learning experience and increased interest in the specialty. An objective assessment of skills or knowledge was not performed in this survey. Additionally, we utilized neither a control nor a comparator group. Besides, our work is purely descriptive. No variability measures or subgroup analyses were performed. Therefore, we cannot claim effectiveness, but only acceptability. Furthermore, our two evaluation items showed a pronounced ceiling effect: 96.6 and 98.0% of students endorsed the two most favorable response categories. Because of this fact, the 6-point scale utilized by us produced no meaningful distributional spread. This bears on the interpretability of the data and the suitability of the instrument for future use.

Another very important point of criticism is the fact that there is a high risk of social desirability bias, whereas the students answered anonymously. The evaluation was conducted within the teaching environment and there was no external validation. Because of these facts, there is a high risk of overinterpretation of the evaluation results gained from the students.

In the future, it would be desirable to conduct a comparative study with different cohorts of medical students using validated evaluation tools, including pre/post assessment and examining objective outcome measures with the aim of continuously improving the quality of student teaching.

## Conclusion

The descriptive study shows that the students evaluated the skills-based practical internship positively, but they do not demonstrate that the teaching concept improves competence or is superior to other formats. We suggest our practice-oriented block internship concept, which focuses on learning skills relevant to the future medical profession, as a possible curriculum for student teaching in the field of gynecology and obstetrics, which could be also performed at other university hospitals. Nevertheless, further studies including objective outcome measures and pre/post comparison are necessary.

## Data Availability

No datasets were generated or analysed during the current study.

## References

[1] Atiomo WU, Casper G, Symonds I, Obermair HM, Gwako G, Vash-Margita A, Sosa C, Kihara A, Ezimokhai M, Fogarty P (2024) A common curriculum in obstetrics and gynecology for medical students globally. Int J Gynaecol Obstet 167(1):191–196 38666747 10.1002/ijgo.15544

[2] Olmes GL, Zimmermann JSM, Stotz L, Takacs FZ, Hamza A, Radosa MP, Findeklee S, Solomayer EF, Radosa JC (2021) Students’ attitudes toward digital learning during the COVID-19 pandemic: a survey conducted following an online course in gynecology and obstetrics. Arch Gynecol Obstet 304(4):957–963 34355284 10.1007/s00404-021-06131-6 PMC8341044

[3] Takacs FZ, Solomayer EF, Hamza A, Juhasz-Böss I, Sklavounos P, Radosa JC, Findeklee S (2020) Conisation course for medical students-experience from a German university hospital. J Turk Ger Gynecol Assoc 21(2):79–83 31612696 10.4274/jtgga.galenos.2019.2019.0126 PMC7294840

[4] Findeklee S, Hamoud BH, Diedrich K, Sima RM, Solomayer EF, Spüntrup C (2024) Training of young medical professionals: implementation of modern training concepts, taking into account the changed framework conditions for training-a pilot project for operational subjects. Arch Gynecol Obstet 309(6):2727–2733 38538859 10.1007/s00404-024-07447-9 PMC11147818

[5] Yu JH, Chang HJ, Kim SS, Park JE, Chung WY, Lee SK, Kim M, Lee JH, Jung YJ (2021) Effects of high-fidelity simulation education on medical students’ anxiety and confidence. PLoS ONE 16(5):e0251078 33983983 10.1371/journal.pone.0251078 PMC8118241

[6] Okuda Y, Bryson EO, DeMaria S Jr, Jacobson L, Quinones J, Shen B, Levine AI (2009) The utility of simulation in medical education: what is the evidence? Mt Sinai J Med 76(4):330–343 19642147 10.1002/msj.20127

[7] Solotke MT, Crabtree J, Encandela J, Vash-Margita A (2020) Establishing a pediatric and adolescent gynecology subinternship for medical students. J Pediatr Adolesc Gynecol 33(2):104–109 31672667 10.1016/j.jpag.2019.10.004

[8] Lerner V, Higgins EE, Winkel A (2018) Re-boot: simulation elective for medical students as preparation bootcamp for obstetrics and gynecology residency. Cureus 10(6):e2811 30116684 10.7759/cureus.2811 PMC6092190

[9] Adams J, Stosch C, Mallmann M, Adams NM, Ludwig S (2025) Bridging the gap: effects of simulation-based OB/GYN training on skills and self-perception in final-year medical students. Front Med (Lausanne) 12:1716282 41476883 10.3389/fmed.2025.1716282 PMC12747925

[10] Duhm L, Wittek A, Walter A, Plöger R, Haverkamp N, Marinova M, Strizek B, Recker F (2025) Exploring undergraduate medical students’ perceptions and career choices in obstetrics and gynecology. Geburtshilfe Frauenheilkd 85(3):333–343 40052015 10.1055/a-2500-0078 PMC11882317

[11] Hamza A, Warczok C, Meyberg-Solomayer G, Takacs Z, Juhasz-Boess I, Solomayer EF, Radosa MP, Radosa CG, Stotz L, Findeklee S, Radosa JC (2020) Teaching undergraduate students gynecological and obstetrical examination skills: the patient’s opinion. Arch Gynecol Obstet 302(2):431–438 32488397 10.1007/s00404-020-05615-1 PMC8595149

[12] Findeklee S, Breitbach GP, Radosa JC, Morinello E, Spüntrup E, Solomayer EF, Spüntrup C (2020) Significant improvement of laparoscopic knotting time in medical students through manual training with potential cost savings in laparoscopy - an observational study. J Turk Ger Gynecol Assoc 21(3):150–155 32517433 10.4274/jtgga.galenos.2020.2020.0019 PMC7495131

